# Mitochondrial Genetic Background Modifies the Relationship between Traffic-Related Air Pollution Exposure and Systemic Biomarkers of Inflammation

**DOI:** 10.1371/journal.pone.0064444

**Published:** 2013-05-23

**Authors:** Sharine Wittkopp, Norbert Staimer, Thomas Tjoa, Daniel Gillen, Nancy Daher, Martin Shafer, James J. Schauer, Constantinos Sioutas, Ralph J. Delfino

**Affiliations:** 1 Occupational and Environmental Medicine Division, Department of Medicine, School of Medicine, University of California Irvine, Irvine, California, United States of America; 2 Department of Epidemiology, School of Medicine, University of California Irvine, Irvine, California, United States of America; 3 Department of Statistics, School of Information and Computer Sciences, University of California Irvine, Irvine, California, United States of America; 4 Department of Civil and Environmental Engineering, Viterbi School of Engineering, University of Southern California, Los Angeles, California, United States of America; 5 University of Wisconsin-Madison, Environmental Chemistry and Technology Program, Madison, Wisconsin, United States of America; Universität Bochum, Germany

## Abstract

**Background:**

Mitochondria are the main source of reactive oxygen species (ROS). Human mitochondrial haplogroups are linked to differences in ROS production and oxidative-stress induced inflammation that may influence disease pathogenesis, including coronary artery disease (CAD). We previously showed that traffic-related air pollutants were associated with biomarkers of systemic inflammation in a cohort panel of subjects with CAD in the Los Angeles air basin.

**Objective:**

We tested whether air pollutant exposure-associated inflammation was stronger in mitochondrial haplogroup H than U (high versus low ROS production) in this panel (38 subjects and 417 observations).

**Methods:**

Inflammation biomarkers were measured weekly in each subject (≤12 weeks), including interleukin-6 (IL-6), tumor necrosis factor-α (TNF-α), C-reactive protein, interleukin-6 soluble receptor and tumor necrosis factor-soluble receptor II. We determined haplogroup by restriction fragment length polymorphism analysis. Air pollutants included nitrogen oxides (NO_x_), carbon monoxide (CO), organic carbon, elemental and black carbon (EC, BC); and particulate matter mass, three size fractions (<0.25 µm, 0.25–2.5 µm, and 2.5–10 µm in aerodynamic diameter). Particulate matter extracts were analyzed for organic compounds, including polycyclic aromatic hydrocarbons (PAH), and *in vitro* oxidative potential of aqueous extracts. Associations between exposures and biomarkers, stratified by haplogroup, were analyzed by mixed-effects models.

**Results:**

IL-6 and TNF-α were associated with traffic-related air pollutants (BC, CO, NO_x_ and PAH), and with mass and oxidative potential of quasi-ultrafine particles <0.25 µm. These associations were stronger for haplogroup H than haplogroup U.

**Conclusions:**

Results suggest that mitochondrial haplogroup U is a novel protective factor for air pollution-related systemic inflammation in this small group of subjects.

## Introduction

Traffic-related air pollution has been shown to be associated with cardiovascular and respiratory morbidity and mortality in many epidemiological studies [1], [2]. These associations hold for both long- and, importantly, short-term exposure-response relations [1]. Increases in short-term air pollutant exposures, with exposure times of hours to days, have been associated with major cardiovascular events such as MI [3], [4] and stroke, as well as cardiovascular hospital admissions and mortality [1]. In addition, there are associations between exposure and subclinical effects such as increases in blood pressure, ST-segment depression and increases in blood biomarkers of cardiovascular risk (reviewed by Brook et al. [1]).

A growing body of research supports the hypothesis that mechanisms behind the morbidity and mortality associations with traffic-related air pollution exposure involve inflammation and oxidative stress induced by potentially pro-oxidant chemical components [1]. Traffic-related particles have been shown to contain redox active chemicals, such as quinones and transition metals, that may be responsible for increases in oxidative stress [5]. Oxidative stress is implicated in progression of coronary artery disease and inflammation, making air pollution-related oxidative stress an important potential cause of disease progression and exacerbation [6]. Previous work by our group has shown that particulate matter (PM) air pollutant exposures, linked to primary products of fossil fuel combustion measured in outdoor home air, were associated with systemic inflammation biomarkers in elderly subjects with coronary artery disease [7]–[9]. These relationships were seen primarily for exposure markers of traffic-related air pollution such as elemental carbon (EC), polycyclic aromatic hydrocarbons (PAH), CO, and NO_x_, and for the quasi-ultrafine (<0.25 µm in diameter) mass fraction of PM (PM_0.25_). Oxidative potential of particle extracts collected near subject homes, as measured by the ability of particle extracts to induce reactive oxygen species (ROS) production in rat alveolar macrophages, was also associated with systemic inflammation [8].

From epidemiological and *in vitro* evidence, mitochondria appear to be a key cellular target for PM air pollution. In a cross-sectional study of steel workers, PM concentration was associated with mitochondrial dysfunction as measured by mitochondrial DNA copy number [10]. Ultrafine particles (diameter <0.1 µm) that are an enriched particle fraction of traffic emissions have been shown to localize to and damage mitochondria in cultured cells [11], while diesel exhaust particles disrupt the inner mitochondrial membrane and cause increases in reactive oxygen species (ROS), H_2_O_2_ and superoxide [12]. Additionally, Soberanes et al. showed that PM_2.5_ exposure in cultured alveolar epithelial cells induces mitochondrial ROS that activates a signaling cascade leading to cell death [13]. Thus, both experimental and human observational studies support the potential role of mitochondrial ROS in pollutant-related pathologies.

Mitochondria are the principle site of oxidative energy metabolism in eukaryotic cells. During normal respiration, mitochondria produce ROS, mostly in the form of superoxide [14], and it is estimated that up to two percent of oxygen consumed by an adult human is converted to mitochondrial superoxide [15]. Overproduction of mitochondrial ROS is associated with early onset and progression of atherosclerosis [16]. Compounding these relationships, the mitochondrial genome contains inherited polymorphisms that define the mitochondrial haplogroups and are proposed to alter the coupling of the respiratory chain [17]. Such alterations affect the level of endogenous ROS production of the mitochondria [18]; more tightly coupled respiratory chains are more efficient, producing more adenosine triphosphate and less heat, but greater amounts of ROS as a byproduct. Haplogroups H, U, JT, and IWX make up approximately 90% of European Caucasians. Compared to equatorial haplogroups, mitochondria of European haplogroups are relatively uncoupled. Within the common European haplogroups, H is relatively tightly coupled; it is associated with increased oxidative damage and increased risk of diseases of aging, such as Parkinson's disease [19], [20]. Less tightly coupled haplogroup U has been shown to be protective of Alzheimer's disease and Parkinson's disease; it has also been associated with increased longevity among Irish and Finnish populations [19]. This protective effect may be due to the decreased ROS production through the relatively uncoupled respiratory chain. Apparent involvement of mitochondria in the cellular responses to pollution exposures suggests that these genetically-determined differences in mitochondrial ROS production may result in different susceptibility to the pro-oxidant effects of air pollutants.

Because haplogroup alters mitochondrial ROS production, we hypothesize that mitochondrial haplogroup will modify our previously reported associations of proinflammatory biomarkers with air pollution exposures [7], [9], [21]. The objective of this study was to test our hypothesis that haplogroup is an effect modifier of the relation between proinflammatory biomarkers and air pollution. Haplogroups H and U are European haplogroups with established clinical and cellular differences and substantially contrasting levels of respiratory chain coupling as described above. Because there is mechanistic reason to expect these specific groups to differ in oxidative stress-related outcomes, only those subjects found to be H or U were included in the main analysis.

## Methods

### Design and population

A cohort panel design was used with repeated measures of outcomes and exposures of subjects living in four retirement communities in the Los Angeles air basin. Due to its unique topography, weather and traffic patterns, this area provides an opportunity to observe air pollution levels that are typically above average versus other urban areas in the United States. Since pollution composition varies in the warm and cool seasons [22], we followed subjects in two seasonal phases for greater contrast in exposure variables. We used repeated measures analysis that allows us to associate outcomes with relatively small, short-term fluctuations in pollution level. Subjects included those studied previously (for details see Delfino et al. [21]). The cohort included 60 adults over age 65 years with coronary artery disease (CAD) confirmed by research team cardiologists, who were current non-smokers. Baseline questionnaires were used to collect information on time-invariant subject characteristics such as age, sex, and comorbidities. The only time-variant subject characteristic we were able to capture was presence of respiratory, urinary tract or other infections during weeks of biomarker measurement.

*Ethics Statement:* All subjects provided written informed consent for participation. This study was performed under the approval of the Institutional Review Board of the University of California, Irvine.

### Biomarker measurement

Peripheral blood was collected into EDTA tubes weekly on Friday afternoons to control for diurnal and daily variation, and plasma was separated, frozen to −80°C on site, and stored at −80°C until assayed. We used 96-well microplate ELISA kits to measure IL-6 and TNF-α [Quantikine HS, R&D Systems, Minneapolis, MN]; high sensitivity CRP [Zymutest; Hyphen BioMed, Neuville-sur-Oise, France]; and soluble cytokine receptors IL-6 soluble receptor (IL-6sR) and TNF-α soluble receptor II (sTNF-RII) [Quantikine R&D Systems]. Twelve IL-6 observations with values greater than 10 pg/mL were reset to 10 pg/mL (upper limit of detection).

### Genotyping

Restriction fragment length polymorphism analysis was performed on all 60 subjects to identify subjects in haplogroups H and U using established restriction fragment length polymorphism analysis [23]. Mitochondrial DNA segments were amplified by PCR and digested using published conditions [19], then separated on 4% agarose stained with ethidium bromide. Fragment patterns were read for H or U designation. Subjects determined to be non-H, non-U were excluded from our main analysis because they represent an unknown mixture of haplogroups.

### Exposure measurement

We measured the concentrations of outdoor air pollutants on site for each of the participating retirement communities during the week preceding each blood collection. Importantly, inclusion criteria were such that participants could not be employed out of the home, improving the accuracy of our exposure measurements. We used standard federal reference methods to measure EPA-regulated criteria gaseous pollutants, hourly NO_2_/NO_x_ and CO. We also measured hourly PM_2.5_ elemental and organic carbon (EC, OC) [OC_EC Analyzer, Model 3F, Sunset Laboratory Inc., Tigard, OR], and black carbon (BC) [Aethalometer, Magee Scientific, Berkeley, CA].

Size-fractionated PM mass concentrations were determined for quasi-ultrafine particles (PM_0.25_), accumulation-mode particles (PM_0.25–2.5_) and coarse mode particles (PM_2.5–10_). We collected samples of PM_0.25_, PM_0.25–2.5_ and PM_2.5–10_ on Teflon filters. Five-day composites were used to determine accumulation-mode and quasi-ultrafine particle organic components described elsewhere [21] (see Table S1). Using Gas Chromatography/Mass Spectrometry we measured polycyclic aromatic hydrocarbons (PAHs), which are largely products of fossil fuel combustion in the study area; hopanes, found in the lubricant oils of diesel and gasoline vehicles and are thus tracers of particles from these vehicles; and organic acids, namely *n*-alkanoic acids between C_14_ to C_30_, which are tracers of photochemically-produced secondary organic aerosols (SOA) in the Los Angeles air basin [24]. A General Electric instrument (Sievers Total Organic Carbon, TOC; GE, Inc.) was used to determine concentrations of water-soluble organic carbon (WSOC), which is a tracer of biomass burning and photochemically-produced SOA [25], [26] in the basin. We estimated secondary WSOC as measured WSOC minus the fraction of WSOC attributed to wood smoke. The latter was estimated as 71% of OC from wood smoke [determined from chemical mass balance (CMB, EPA-CMB8.2) model output] [27]. Secondary WSOC was then converted to SOA using methods described by us in a CMB analysis [28].

Aqueous particle extracts from PM_0.25–2.5_ and PM_0.25_ were also tested for *in vitro* capacity to induce production of reactive oxygen species (ROS) using a rat alveolar macrophage assay described elsewhere [29].

### Statistical Analysis

Mean values of inflammatory biomarkers for H versus U haplogroups were compared by Student's t-test with unequal variances. Because we expect infectious processes to generate inflammation at high levels that would obscure potentially subtle effects of haplogroups and pollutants, biomarker observations were excluded for 33 observations when subjects experienced infections leaving 417 observations between H and U subjects.

To test our hypothesis that haplogroup is an effect modifier of the relationship between pollutant exposure and inflammatory biomarker levels, we used generalized linear mixed models to regress biomarkers on individual pollutants, and included a product term between haplogroup and pollutant. Because the study was a longitudinal repeated measures study, subjects had no meaningful baseline data. However, we incorporated random subject intercepts in the model, such that the results reflect within-subject changes in biomarker as a function of changes in pollutant. As the original cohort panel was not powered to determine effect modification, a nominal *p*-value<0.1 for the product term was considered to be evidence of significant interaction to avoid increased type II error rates at this early stage of investigation. An autoregressive-1 covariance structure between random within-person errors in the dependent variables provided the best model fit. To standardize comparisons for different pollutants, we examined associations for an interquartile range increase in each pollutant. Pollutant exposures were averaged over time periods preceding biomarker measurement, with each cumulative averaging period (1 to 9 days leading up to biomarker measurement) tested in its own model. Exposure variables were mean-centered, as done in our previous analyses [7]–[9] to generate parameter estimates for the within-study period, within-subject effect of exposure. Residuals were analyzed for a subset of models to identify highly influential points. Since time-invariant within-subject characteristics are unlikely to act as confounders in a relationship between outcome and air pollution, these variables were left out of the pollutant models. Additionally, when these variables were included in exploratory models of inflammation predicted by haplogroup, the haplogroup coefficient was stable indicating they have little influence on the relationship of interest (See Text S1, Table S2).

In Supporting Information (Text S1, Table S2), we present an exploratory analysis of the direct relationship between haplogroup and inflammation. This analysis is considered exploratory due to the small number of subjects and thus fixed predictors. We also present mixed model analyses of pollutant-Haplogroup interactive effects including the “Other” group of non-H, non-U subjects (Figure S1, Figure S2).

## Results

### Descriptive data

From our cohort of 60 subjects, 27 were determined to belong to haplogroup H and 9 belong to haplogroup U. Non-H, non-U subjects have mitochondria from any other haplogroup. Maternal lineage alone determines mitochondrial haplogroup, independent from nuclear DNA inheritance and phenotype. Thus, these individuals represent an unknown mixture of global coupling efficiencies despite European Caucasian heritage. Hence, we restrict the analysis to the H and U groups that have established contrasts biochemically, and clinically. Table 1 shows the subject characteristics for this subset of our cohort. Haplogroup H individuals, on average had 1.1 pg/mL higher IL-6 versus those in haplogroup U (p<0.001) in a non-parametric Satterthwaite t-test. Table 2 lists descriptive statistics for pollutants in our analysis.

**Table 1:** Subject Characteristics by Haplogroup.

	All Subjects	Haplogroup H	Haplogroup U
Mean (SD) or N (%)	n = 36	n = 27	n = 9
Age	84.08 (5.63)	84.48 (5.59)	82.89 (5.93)
Body Mass Index	26.98 (3.98)	26.74 (4.1)	27.67 (3.7)
Male	21 (58.33)	15 (55.56)	6 (66.67)
*Smoking status*			
Never	21 (58.33)	15 (55.56)	6 (66.67)
Past Smokers	14 (38.89)	11 (40.74)	3 (33.33)
Missing	1 (2.78)	1 (3.70)	0 (0)
*Cardiovascular History*			
Myocardial Infarction	11 (30.56)	8 (29.63)	3 (33.33)
Congestive heart failure	6 (16.67)	4 (14.81)	2 (22.22)
Hypertension	24 (66.67)	18 (66.67)	6 (66.67)
High cholesterol (by history)	25 (69.44)	17 (62.96)	8 (88.89)
*Other medical history*			
Diabetes	6 (16.67)	4 (14.81)	2 (22.22)
Chronic obstructive pulmonary disease	2 (5.56)	2 (7.40)	0 (0)
Asthma	2 (5.56)	0 (0)	2 (22.22)
*Medications*			
ACE inhibitors	12 (33.33)	11 (40.74)	1 (11.11)
HMGCoA Reductase Inhibitors	23 (63.89)	17 (62.96)	6 (66.67)
Platelet Inhibitors	15 (41.67)	12 (44.44)	3 (33.33)
*Biomarkers of Inflammation*			
C-reactive Protein (ng/mL)	2245 (2238)	2385 (2467)	1824 (1372)
Tumor necrosis factor-α (pg/mL)	1.71 (1.21)	1.78 (1.26)	1.51 (1.06)
Soluble TNFα receptor II (pg/mL)	3541 (1364)	3669 (1469)	3156 (952)
Interleukin-6 (pg/mL)*	2.40 (1.76)	2.69 (1.91)	1.52 (0.75)
Soluble IL6 receptor (pg/mL)	39967 (11160)	40339 (12188)	38853 (7761)

* p<0.001 comparing H vs. U.

**Table 2:** Outdoor air pollutant concentrations in the retirement communities.

Air Pollutant Exposures^a^	Mean (SD)	Median	IQR	Min/Max
Black Carbon (µg/m^3^)	1.68 (7.86)	1.57	0.99	0.30/5.11
Elemental Carbon (µg/m^3^)	1.50 (0.62)	1.42	0.81	0.24/3.94
Organic Carbon (µg/m^3^)	8.53 (4.55)	7.11	6.30	2.32/27.26
NO_x_ (ppb)	45.35 (31.05)	39.26	38.83	3.70/188.00
CO (ppm)	0.54 (0.30)	0.49	0.43	0.01/1.68
O_3_ (ppb)	27.13 (11.76)	25.65	16.96	6.17/76.35
Particle Mass				
PM_0.25_ (µg/m^3^)	9.77 (4.12)	9.33	5.28	2.46/30.05
PM_0.25–2.5_ (µg/m^3^)	11.37 (9.40)	9.05	10.36	0.98/66.77
PM_2.5–10_ (µg/m^3^)	9.38 (4.98)	9.02	7.00	0.30/24.63
5-day composite of PM_2.5_ components				
PAH (ng/m^3^)	0.49 (0.16)	0.43	0.16	0.33/1.01
Hopanes (ng/m^3^)	0.40 (0.29)	0.34	0.32	0.10/1.45
Organic Acids (µg/m^3^)	41.60 (37.17)	28.18	42.57	9.74/149.82
WSOC (µg/m^3^)	0.46 (0.27)	0.40	0.30	0.09/1.37
Macrophage ROS				
PM_0.25–2.5_ (µg Zymosan equivalents/m^3^)	6264.04 (3184.18)	4972.13	5052.00	1646.96/13073.52
PM_0.25_ (µg Zymosan equivalents/m^3^)	36331.77 (34893.48)	20142.30	35414.00	2585.33/147217.37
Temperature (°C)	18.65 (5.94)	18.46	8.64	1.46/33.08

a During the 47 weeks of air monitoring exposure data presented are for daily averages except for the 5-day composites of particle filters extracted for the measurements of organic components and macrophage ROS.

Pollutant correlations have been described in a previous publication by our group (see Delfino et al. [7], [9], [21]), but generally we observed strong correlations between the traffic-related air pollutants (EC, BC, NO_x_, and CO), which themselves were negatively correlated with O_3_ [7].

### Regression models

We found significant associations of IL-6 and TNF-α with multiple markers of traffic related air pollution. Although some consistent associations were observed for the other biomarkers, confidence limits were wider (see Table S3). To simplify presentation, we show a subset of IL-6 and TNF-α models in relation to air pollutants with averaging times of one, two, three or five days; results for seven and nine-day averages were comparable to 5-day averages. These models concern the effects of temporal variation in air pollutant levels on temporal variation in biomarkers and thus do not adjust for individual time-invariant characteristics (inter-individual variations in subject characteristics are partly accounted for by the random intercepts).

The analysis focused on a random effects model that included a random intercept term to account for differential mean biomarker levels across individuals. In this modeling framework, we are considering the within-subject change in biomarker as a function of changes in pollution, conditional upon each subject's latent random intercept. We have performed residual diagnostics for functional form and these have not revealed departures from linearity in the association between pollution and IL-6 or TNF- α. Therefore, conditional on the random intercept, mean differences in the distribution of biomarkers by haplogroup would not have induced the effect modification that is observed in our study.

**Figure 1:**
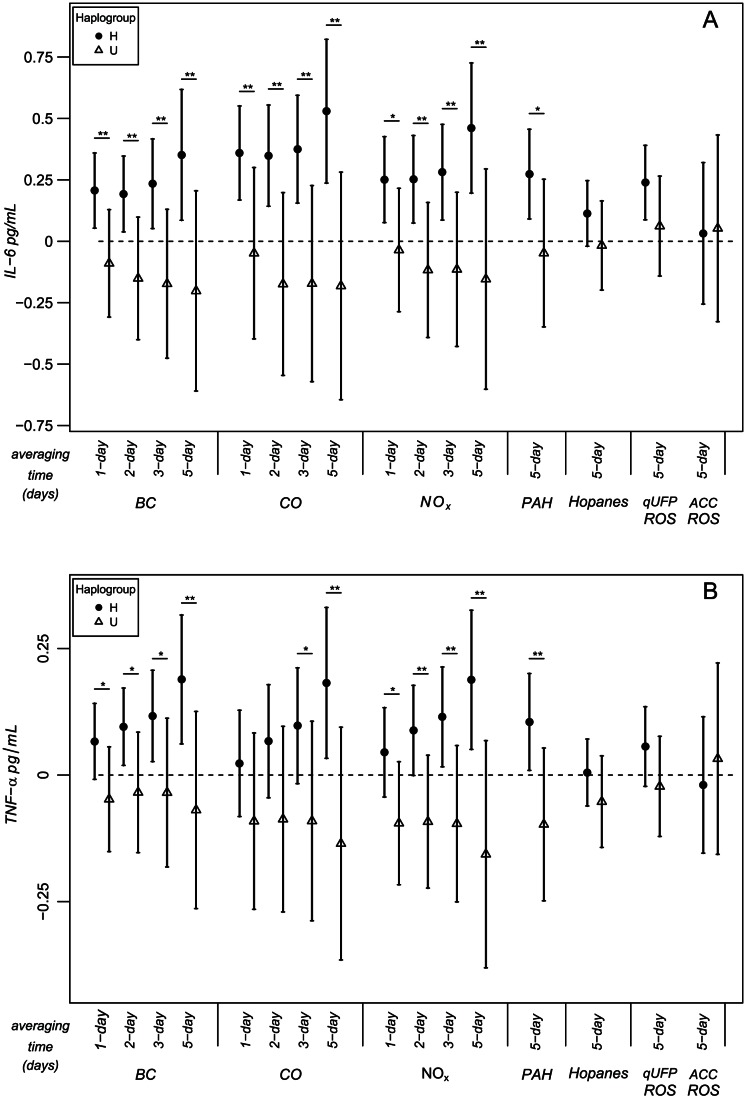
Associations of biomarkers with traffic-related air pollutants: effect modification by mitochondrial haplogroups H and U. Expected change in IL-6 (A) and TNF-α (B) (coefficient and 95% CI) corresponds to an IQR increase in air pollutant exposure (Table 2). p-value for interaction between haplogroup H and U: ^*^p≤0.1. ^**^p≤0.05.

**Figure 2:**
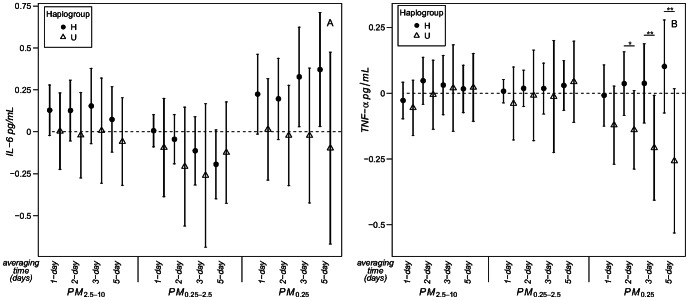
Associations of biomarkers with size-fractionated PM: effect modification by mitochondrial haplogroups H and U. Expected change in IL-6 (A) and TNF-α (B) (coefficient and 95% CI) corresponds to IQR increase in air pollutant exposure (Table 2). p-value for interaction between haplogroup H and U: ^*^p≤0.1. ^**^p≤0.05.

For markers of traffic-related air pollution (PAH, BC, CO, and NO_x_), increases in IL-6 and TNF alpha were associated with increases in pollutant levels. Figure 1 shows stratified estimates for H and U haplogroups for each of these pollutants. We found similar significant associations for EC, and similar, though nonsignificant, trends for PM_0.25_ (quasi-ultrafine particles, qUFP) and coarse mode particulate matter (PM_2.5–10_) (Figure 2). The stratified pollutant-biomarker associations were only significantly positive among the subjects with haplogroup H. For example, in a model with among the greatest differences in association, an interquartile range (IQR) increase of 0.51 ppm in 5-day average CO was associated with IL-6 increases in H subjects of 0.529 pg/mL (p = 0.0004) and −0.182 pg/mL (p = 0.441) in U subjects (interaction term p = 0.010). The same IQR increase in CO was associated with a TNF-α increase in H subjects of 0.182 pg/mL (p = 0.017) and a nonsignificant decrease of 0.135 in U subjects (interaction term p = 0.021). Most air pollutant associations with IL-6 among H participants were significant for all averaging times, but were increasingly strong with longer average times. None of the one-day average pollutant levels were significantly associated with TNF-α level, however the strength and significance of the association increased with increasing averaging times. It is important to note that significant interaction terms (p<0.1) indicate different effects in the groups, not just observed effects in H and non-significant effects in U.

We examined polycyclic aromatic hydrocarbons (PAH) as a tracer of primary combustion emissions because these are established products of incomplete combustion of petroleum products. We found that PAH was a significant predictor of biomarker level, but only for those subjects with haplogroup H mitochondrial DNA (Figure 1). For an IQR increase in total PAH of 0.560 ppb, IL-6 increased by 0.273 pg/mL (p = 0.003) among H subjects, versus a decrease of 0.047 pg/mL among subjects in haplogroup U (interaction p = 0.072). Similarly, an IQR increase in total PAH of 0.560 ppb was associated with 0.105 pg/mL higher TNF-alpha level among haplogroup H subjects (p = 0.032), versus a nonsignificant 0.09 pg/mL decrease (p = 0.205) among U subjects (interaction p = 0.73). The secondary (photochemical) air pollutant markers, such as ozone, WSOC and organic acids, had no significant associations with either biomarker, except for WSOC, which was associated with a significantly higher level of TNF-alpha among H's. We also tested the WSOC fraction attributable to wood smoke and found no significant associations or interactions. Because the major source of air pollution in the Los Angeles air basin is traffic, this pollutant fraction was minor in comparison, thus effects shown in other studies of pollution from wood burning were not expected to be present here.

We observed positive associations of IL-6 with 3-day and 5-day PM_0.25_ averages that were significant among the haplogroup H subjects (Figure 2). TNF-alpha was positively associated qUFP as well and, while the stratified estimates for haplogroup H were not significant, the interaction terms were, indicating stronger associations for H than for U subjects. The other particle size fractions were not associated with either TNF-alpha or IL-6.

In this study, we tested many interactions, hence multiple testing bias is a concern and could increase the likelihood of type I error. No direct adjustment for multiple comparisons has been made due to the complex and unknown correlation among the tests performed [30]. However, we note that out of 164 tests of interaction, for all pollutants and averaging times tested in interactions with haplogroup as predictors of TNF-α and IL-6, 63 tests (38%) were significant at the selected 10% level while 16.4 significant test were expected due to chance, and 39 tests (24%) were significant at the 5% level while 8.2 significant tests were expected due to chance. For the other biomarker outcomes, the number of significant tests of interaction was no greater than that expected by chance.

## Discussion

In our cohort of elderly individuals with CAD, we found that mitochondrial haplogroup was an effect modifier of the relationship of systemic biomarkers of inflammation with traffic-related air pollution exposures (BC, PAH, CO and NO_x_) and with both quasi-ultrafine particle mass and oxidative potential. Exposures among those in haplogroup H are associated with greater increases in systemic inflammation compared to haplogroup U, supporting our hypothesis that intrinsic oxidative stress from mitochondrial ROS could increase susceptibility to the adverse effects of air pollutants. Haplogroup U has been shown to be protective of other oxidative stress-related outcomes but, to our knowledge, this result represents the first epidemiological evidence that inherited mitochondrial variation modifies air pollution exposure-response relations.

Interleukin-6 (IL-6) has been used as a biomarker of systemic inflammatory response to air pollution exposure in previous studies of human subjects [1], including our own [7]–[9]. ROS-signaling by inflammatory cytokines (e.g. TNF-alpha) is an important inflammatory pathway [31]. Mitochondrial ROS has been implicated in inflammatory cytokine production, providing a possible link between ROS-driven and inflammatory diseases [32]. Studies in cultured human cells have shown that exposure to particulate matter increases mitochondrial ROS [33]–[35], and these mitochondrial ROS increases cause increased IL-6 gene expression [34], [35]. Conversely, inhibition of mitochondrial ROS reduces inflammatory cytokine production in response to inflammatory stimulus [36]. Urban PM has an ability to directly induce oxidative stress and inflammatory responses via the endogenous production of ROS by cells due to pro-oxidant chemicals [11]. We have shown here that mitochondrial haplogroup, which is known to alter mitochondrial ROS production, is an effect modifier of associations between plasma IL-6 and air pollution in human subjects, results that are corroborated by *in vitro* research. The potential clinical importance of this finding is that adverse health outcomes are associated with increased IL-6 levels (see Singh and Newman [37] for a review of IL-6 related morbidity in aging populations). Elevated levels of IL-6 are associated with increased risk of cardiovascular disease [38], and mendelian randomization studies suggest a causal role for IL-6 but not CRP [39], which was not associated with air pollutants or modified by mitochondrial haplogroup (Table S2). In a population with existing CAD, Fisman et al. (2006) found that a 1 pg/mL increase in IL-6 was associated with 70% increased odds of acute MI or sudden death [40]. Thus, despite the small number of subjects, our high number of repeated measures allowed us to find significant differences in IL-6 between haplogroups H and U (1.17 pg/mL, Table 1) that may be of clinical significance.

Our results show stronger effect modification by haplogroup on pollutant associations with IL-6 than on those with TNF-α, though both have multiple significant associations. TNF-α and ROS have a bidirectional relationship, wherein TNF-α increases ROS production via signaling through TNFR1, but ROS can increase TNF-α levels [41], possibly through NFκB [42]. Since TNF-α increases expression of IL-6, which then suppresses TNF-α, the increases in TNF-α associated with pollutant exposure may have been blunted, decreasing our ability to tease out the association of haplogroup with this cytokine. However, we did observe expected effect modification of the relation of established traffic-related air pollutants with TNF-α.

Because traffic exhaust is an important source of ultrafine particles in urban areas, our findings for qUFP are of particular relevance in the Los Angeles region. Although the interaction term p-value is not p<0.1 for qUFP and haplogroup, the differences in association are consistent with our other traffic-related air pollutant findings and adds to growing evidence that traffic pollution contributes to the risk of adverse cardiovascular outcomes [1]. We found effect modification of the association between biomarkers and the oxidative potential of qUFP (macrophage ROS induction by particle extracts), a novel exposure metric. This result suggests that stronger associations between air pollution and systemic inflammation in Haplogroup H versus U could be attributable to the oxidative potential of particles and consequent oxidative stress. The present study shows fewer pollutants with significant associations than our previous analyses; this could be due to the loss of power in the smaller subset stratified by *a priori*-selected haplogroups.

There are several limitations to our study. Mitochondrial haplogroups H and U are gross designations, macrohaplogroups, each of which contain additional recent mutations that further modulate the relationships between haplogroup and oxidative stress. For example, haplogroup H is defined by a T to C transition at position 7028 of the mitochondrial genome, subunit 1 of the cytochrome c oxidase, complex IV of the respiratory chain and it is known that carbon monoxide inhibits complex IV [43]. H individuals would be expected to respond differently to such inhibition, though subgroups may have additional modulating SNPs. Our results show H individuals have significantly higher levels of inflammatory biomarkers associated with CO exposure versus U individuals. However, the low level of CO exposure in the present study is taken to represent other causal pollutants in the mixture of traffic-related air pollution. Future studies could demonstrate additional, subtle effects of subgroup-defining mutations, with larger sample sizes needed to give adequate representation of these subgroups. However, we are still able to see the effects of these founding mutations in our cohort. In addition to analyzing only macrohaplogroups, we were limited by a small sample size for those in haplogroup U; this may have been responsible for the wide confidence intervals and nonsignificance for estimates in this group. However, given that the interaction terms were significant for many of the relationships shown, it is likely that increasing the sample size would strengthen our findings rather than generating significant positive estimates for the U haplogroup.

This study was also limited because we could not account for other genetic or lifestyle differences in antioxidant pathways. It is possible that variations in other antioxidant defenses, such as increased superoxide dismutase activity, could account for a relatively decreased response the U group versus the H group. However, the likelihood that only those in group U possessed increased antioxidant defenses from the nuclear genome or diet is low. Since mitochondria are inherited maternally independent from nuclear DNA, there is no reason to believe haplogroup SNPs are linked to particular nuclear genotypes or to diet. We also were unable to capture time-varying subject characteristics, such as daily physical activity levels. It is known that IL-6 levels can be altered by exercise [44], and exercise may be associated with weather or pollutant level; however it is unknown if these behaviors are associated with haplogroup. Future work is needed to investigate how time-varying characteristics such as diet and exercise may interact with haplogroup and environmental exposures.

Our results were for a small elderly population of largely European descent; hence, we cannot generalize results to younger, healthier, or other populations. However, restriction to this small, homogeneous group is also a strength of the study because, being restricted to two European haplogroups with known effects on oxidative stress, the present results are more likely to reflect the mitochondrial influence on associations between systemic inflammation and air pollution. Since the principles underlying the mitochondrial effects on oxidative stress and inflammation are independent of demographics, similar effects would be expected for other comparisons across haplogroups within and between other races.

## Conclusions

Our results suggest that, in a small cohort of elderly adults with CAD, mitochondrial haplogroup U may have lower susceptibility to adverse effects of traffic-related air pollution compared to haplogroup H. These results support the hypothesis that relatively small differences in mitochondrial coupling efficiency, which alter the cellular oxidative burden, may alter responses to exogenous inducers of oxidative stress, such as traffic-related air pollution. This potentially important genetic risk factor has not been previously assessed in environmental epidemiological studies. While existing research supports the role of haplogroup in disease risk, several recent studies have shown differences in treatment outcomes by haplogroup [45]–[47]. Additionally, *in vitro*, cytoplasmic hybrid cells show haplogroup differences in response to oxidative stress with antioxidant treatment [48]. This is relevant to advancing the field of personalized medicine since such studies indicate there is potential value in tailoring interventions based on mitochondrial haplogroup. For example, antioxidant treatment in those with greater intrinsic oxidative stress may help ameliorate the proinflammatory effects of environmental pro-oxidant chemicals such as those in traffic-related air pollution. More importantly, this work adds to the growing body of public health knowledge supporting further efforts in promoting clean air that can decrease exposures for everyone. We have shown that mitochondrial haplogroup may be a novel risk factor for air pollution associated-inflammation. Further research is needed to more fully characterize its role, which may allow for targeted measures to decrease or prevent adverse cardiovascular outcomes in exposed populations.

## Supporting Information

Text S1 Exploratory analysis of IL-6 and TNF-α method and results
(DOCX)

Table S1 Exposure variable - Selected Organic Components from particulate matter
(DOCX)

Table S2 Exploratory analysis of IL-6 and TNF-α results table
(DOCX)

Table S3 Analysis of additional Biomarkers: TNFRII, IL6sR, CRP
(DOCX)

Figure S1 Associations of biomarkers with traffic-related air pollutants for haplogroups H, U and Other
(DOCX)

Figure S2 Associations of biomarkers with size-fractionated PM for haplogroups H, U and Other
(DOCX)
