# Supplementary material for: Mitochondrial Genetic Background Modifies the Relationship between Traffic-Related Air Pollution Exposure and Systemic Biomarkers of Inflammation
Source: PLoS One. 2013 May 23;8(5):e64444. doi: 10.1371/journal.pone.0064444 (PMC3662686; doi:10.1371/journal.pone.0064444)
Supplement: Text S1 — Exploratory analysis of IL-6 and TNF-α method and results (DOCX) [file pone.0064444.s001.docx]

**Text S1**

***Exploratory Analysis Method***

We performed an exploratory analysis of the relationship between haplogroup and inflammation. A backward model selection approach was used to build a model estimating the association of each biomarker with haplogroup and the other time-invariant subject characteristics. Subject characteristics analyzed here were those with established associations with inflammatory processes (see Table 1, main text). Each characteristic was used separately to predict biomarker level in a generalized linear mixed model using random intercepts for each subject. Models accounted for correlation within each subject (produced by repeated biomarker measurements) by estimating random effects for each individual, nested within group (retirement community) and phase (cool or warm season). We ran full model including only predictors whose individual association with the biomarker of interest was significant at p=0.05. Predictors were eliminated from the full model sequentially when p-values were more than 0.05, with the haplogroup variable forced into the model regardless of p-value. We analyzed the influence of individual points by selectively removing them from the dataset, running the model, then evaluating any changes in parameter estimates. We evaluated confounding by running two-predictor models with haplogroup and each other variable to determine the extent of change in the haplogroup estimate. We also evaluated interaction terms between haplogroup and each predictor in our final model to look for possible effect modification by haplogroup.

***Results of Exploratory regression analysis of haplogroup and subject characteristics***

Supporting Information Table S2 shows regression coefficients for the full and final models of IL-6 and TNF-α from our exploratory analysis. From the IL-6 model haplogroup H, current congestive heart failure (CHF), prior cerebrovascular accident (CVA), and prior smoking are significant predictors of IL-6 in our cohort. After adjusting for other variables, individuals in haplogroup H had an average IL-6 level that was 34% higher than individuals in haplogroup U (p<0.001). An influence analysis showed three individual subjects with Cook's D greater than 1. Each of these subjects was removed one at a time from the dataset and the model was run. Removal altered neither the direction nor the significance of the haplogroup coefficient. Our two-predictor models with haplogroup and each of the other covariates showed that the inclusion of any of the other predictors did not alter the sign of the haplogroup estimate. When we ran models with interaction terms, the haplogroup H * CHF interaction was nonsignificant. For each of the other two predictors, the interaction was significant. In all models, haplogroup H primary effect remained significant.

In an unadjusted model of TNF- α predicted by Haplogroup, TNF- α is 7% higher among those in haplogroup H versus haplogroup U, but this effect is not significant (p=0.110). However, because this was the variable of interest for the repeated measure study, haplogroup was retained in the backward selection. In our final model, the plasma level of TNF- α is significantly predicted by haplogroup H, male gender, current statin use, a history of hypertension, a history of myocardial infarction (MI), prior CVA, adult onset diabetes mellitus, and prior smoking (Table S2). After adjusting for other covariates, individuals in haplogroup H had TNF- α levels that were 7% higher than those in haplogroup U (p = 0.04). Four observations had Cook's D greater than 1 and were evaluated to determine the strength of their influence. Upon removal of two of these points, the haplogroup coefficient became nonsignificant, though the direction of the coefficient did not change.

Our two-predictor models to test confounding showed that none of the other predictors altered the direction of the haplogroup effect. In four of the models tested (male gender, statin use, diabetes, and prior smoking), the interaction terms were significant.

Overall, models tested showed that within our cohort of elderly individuals with CAD, those in haplogroup H generally have higher levels of biomarkers of systemic inflammation compared to those in haplogroup U.
