# Supplementary material for: Mitochondrial Genetic Background Modifies the Relationship between Traffic-Related Air Pollution Exposure and Systemic Biomarkers of Inflammation
Source: PLoS One. 2013 May 23;8(5):e64444. doi: 10.1371/journal.pone.0064444 (PMC3662686; doi:10.1371/journal.pone.0064444)
Supplement: Table S1 — Exposure variable - Selected Organic Components from particulate matter (DOCX) [file pone.0064444.s002.docx]

**Table S1. Selected organic components measured in quasi-ultrafine mode (PM_0.25_) and accumulation mode PM (PM_0.25-2.5_).**

| **Low molecular weight PAH (2-3 ring)** | **Hopanes** |
| --- | --- |
| Fluoranthene | 17α(H)-22,29,30-Trisnorhopane |
| Acephenanthrylene | 17β(H)-21A(H)-30-Norhopane |
| Pyrene | 17α (H)-21β (H)-Hopane |
| Benzo(ghi)fluoranthene | 22S-Homohopane |
| Benz(a)anthracene | 22R-Homohopane |
| Chrysene | 22S-Bishomohopane |
|  | 22R-Bishomohopane |
| **Medium molecular weight PAH (4 ring)** | 22S-Trishomohopane |
| Benzo(b)fluoranthene | 22R-Trishomohopane |
| Benzo(k)fluoranthene |  |
| Benzo(j)fluoranthene | **Selected organic acids** |
| Benzo(e)pyrene | n-Dodecanoic acid |
| Benzo(a)pyrene | n-Tetradecanoic acid |
|  | n-Pentadecanoic acid |
| **High molecular weight PAH (5-6 ring)** | n-Hexadecanoic acid |
| Indeno(1,2,3-cd)pyrene | n-Heptadecanoic acid |
| Benzo(ghi)perylene | n-Octadecanoic acid |
| Dibenz(ah)anthracene | Palmitoleic acid |
| Picene | Oleic acid |
| Coronene | Phthalic acid |
|  |  |
