# Supplementary material for: Mitochondrial Genetic Background Modifies the Relationship between Traffic-Related Air Pollution Exposure and Systemic Biomarkers of Inflammation
Source: PLoS One. 2013 May 23;8(5):e64444. doi: 10.1371/journal.pone.0064444 (PMC3662686; doi:10.1371/journal.pone.0064444)
Supplement: Table S2 — Exploratory analysis of IL-6 and TNF-α results table (DOCX) [file pone.0064444.s003.docx]

**Table S2. Exploratory Analysis of Biomarker level by Haplogroup**

| **IL-6 Estimate (95%CI)** | | |
| --- | --- | --- |
| Predictor | Full model**^a^** | Final model**^a^** |
| Haplogroup H | 1.216 (0.821, 1.611) | 1.286 (0.904, 1.668) |
| Body mass index | 0.095 (0.043, 0.147) | 0.081 (0.029, 0.133) |
| Current congestive heart failure | 0.838 (0.26, 1.417) | 1.465 (0.989, 1.941) |
| History of cerebrovascular accident | 0.92 (0.378, 1.462) | 1.144 (0.603, 1.685) |
| Adult onset diabetes mellitus | -0.391 (-1.015, 0.232) | -0.59 (-1.136, -0.044) |
| Age | 0.021 (-0.014, 0.057) |  |
| History of hyperlipidemia | -0.456 (-0.887, -0.024) |  |
| History of hypertension | 0.292 (-0.086, 0.672) |  |
| Current angina | 0.801 (0.364, 1.239) |  |
| **TNF-α Estimate (95%CI)** | | |
| Predictor | Full model**^a^** | Final model**^a^** |
| Haplogroup H | 0.282 (0.048, 0.516) | 0.324 (0.1, 0.547) |
| Gender | 0.905 (0.674, 1.136) | 0.88 (0.653, 1.107) |
| Statin use | -0.499 (-0.775, -0.222) | -0.572 (-0.82, -0.324) |
| History of hypertension | -0.604 (-0.862, -0.346) | -0.623 (-0.88, -0.367) |
| History of myocardial infarction | 1.208 (0.966, 1.45) | 1.238 (1.001, 1.475) |
| History of cerebrovascular accident | 1.198 (0.869, 1.526) | 1.234 (0.911, 1.558) |
| Adult onset diabetes mellitus | -0.976 (-1.306, -0.647) | -1.017 (-1.339, -0.694) |
| Prior smoking | 0.624 (0.402, 0.847) | 0.622 (0.399, 0.845) |
| History of hyperlipidemia | -0.17 (-0.455, 0.114) |  |

^a^Results from generalized linear mixed model with multiple measurements of biomarkers, described in Supporting Information Text S1 "Exploratory analysis method and results."
