# Supplementary material for: Mitochondrial Genetic Background Modifies the Relationship between Traffic-Related Air Pollution Exposure and Systemic Biomarkers of Inflammation
Source: PLoS One. 2013 May 23;8(5):e64444. doi: 10.1371/journal.pone.0064444 (PMC3662686; doi:10.1371/journal.pone.0064444)
Supplement: Table S3 — Analysis of additional Biomarkers: TNFRII, IL6sR, CRP (DOCX) [file pone.0064444.s004.docx]

**Table S3. Associations of CRP, IL-6sR and sTNFRII with Outdoor Air Pollutants**

|  |  | **CRP**(95% CI)^a^ | | | **Interaction p-value**^b^ |
| --- | --- | --- | --- | --- | --- |
| **Air Pollutant Exposures** | | **All Subjects** | **Haplogroup H** | **Haplogroup U** |  |
| Black Carbon | 1-day | -4(-259,250) | 45(-250,341) | -113(-536,310) | 0.522 |
|  | 2-day | -116(-377,144) | -60(-359,239) | -267(-749,215) | 0.461 |
|  | 3-day | -191(-499,117) | -152(-505,202) | -303(-888,283) | 0.657 |
|  | 5-day | -164(-608,279) | -1(-515,512) | -569(-1356,218) | 0.221 |
| Elemental Carbon | 1-day | -2(-323,320) | 45(-321,411) | -120(-697,456) | 0.618 |
|  | 2-day | -210(-567,147) | -105(-492,282) | -696(-1495,103) | 0.181 |
|  | 3-day | -79(-414,256) | -21(-394,351) | -283(-979,412) | 0.504 |
|  | 5-day | 4(-407,415) | 96(-360,552) | -364(-1261,533) | 0.363 |
| Organic Carbon | 1-day | -432(-1118,254) | -443(-1271,386) | -411(-1610,788) | 0.966 |
|  | 2-day | -740(-1575,95) | -750(-1747,246) | -714(-2234,806) | 0.969 |
|  | 3-day | -745(-1620,130) | -742(-1794,309) | -752(-2320,817) | 0.992 |
|  | 5-day | -468(-1375,439) | -390(-1501,721) | -627(-2193,940) | 0.808 |
| NO_X_ | 1-day | 224(-62,511) | 267(-68,602) | 138(-345,620) | 0.651 |
|  | 2-day | 44(-251,339) | 98(-246,442) | -82(-613,449) | 0.566 |
|  | 3-day | 42(-281,365) | 51(-325,427) | 32(-575,638) | 0.956 |
|  | 5-day | 90(-355,535) | 164(-351,679) | -103(-973,768) | 0.602 |
| Carbon Monoxide | 1-day | 168(-181,518) | 160(-227,548) | 197(-492,886) | 0.924 |
|  | 2-day | -34(-404,337) | -7(-423,409) | -123(-857,610) | 0.78 |
|  | 3-day | -42(-423,339) | -57(-486,372) | 12(-766,789) | 0.878 |
|  | 5-day | 47(-447,541) | 88(-490,666) | -54(-968,859) | 0.793 |
| O_3_ | 1-day | -88(-522,346) | -91(-590,409) | -81(-961,800) | 0.984 |
|  | 2-day | -274(-814,266) | -324(-951,304) | -125(-1205,955) | 0.755 |
|  | 3-day | -309(-840,222) | -357(-974,261) | -174(-1197,849) | 0.761 |
|  | 5-day | -240(-799,318) | -355(-1001,291) | 65(-959,1089) | 0.486 |
| Particle Mass |  |  |  |  |  |
| PM_0.25_ | 1-day | 91(-287,469) | 142(-331,615) | 11(-587,609) | 0.729 |
|  | 2-day | 73(-329,475) | 167(-347,681) | -74(-712,565) | 0.562 |
|  | 3-day | -18(-530,494) | 87(-540,715) | -224(-1089,641) | 0.562 |
|  | 5-day | -156(-741,429) | -26(-691,639) | -550(-1657,557) | 0.411 |
| PM_0.25-2.5_ | 1-day | -74(-253,106) | -51(-240,138) | -267(-823,288) | 0.469 |
|  | 2-day | -194(-471,82) | -148(-450,155) | -440(-1132,252) | 0.446 |
|  | 3-day | -419(-790,-49) | -324(-732,84) | -859(-1730,12) | 0.274 |
|  | 5-day | -304(-630,22) | -284(-678,110) | -349(-932,234) | 0.855 |
| PM_2.5-10_ | 1-day | -89(-332,154) | -57(-340,227) | -164(-584,257) | 0.666 |
|  | 2-day | -152(-466,161) | -82(-457,293) | -300(-836,236) | 0.502 |
|  | 3-day | -209(-586,169) | -197(-649,256) | -237(-874,399) | 0.916 |
|  | 5-day | -182(-484,120) | -99(-471,273) | -342(-846,162) | 0.441 |
| 5-day composite components | |  |  |  |  |
| PAH |  | 7(-300,314) | 25(-335,386) | -33(-622,557) | 0.869 |
| Organic Acids |  | -117(-424,191) | 56(-296,409) | -631(-1243,-18) | 0.057 |
| WSOC |  | -91(-434,252) | -49(-439,341) | -213(-844,417) | 0.65 |
| Macrophage ROS |  |  |  |  |  |
| PM_0.25-2.5_ |  | -281(-734,171) | -280(-843,283) | -286(-1042,471) | 0.99 |
| PM_0.25_ |  | 197(-43,438) | 157(-141,456) | 279(-119,677) | 0.627 |

**Table S3 (cont.)** **Associations of CRP, IL-6sR and sTNFRII with Outdoor Air Pollutants**

|  |  | **IL6sR** (95% CI)^a^ | | | **Interaction p-value**^b^ |
| --- | --- | --- | --- | --- | --- |
| **Air Pollutant Exposures** | | **All Subjects** | **Haplogroup H** | **Haplogroup U** |  |
| Black Carbon | 1-day | -790(-2159,580) | -1621(-3229,-13) | 963(-1279,3205) | 0.054 |
|  | 2-day | -927(-2361,508) | -1329(-2993,335) | 118(-2479,2714) | 0.345 |
|  | 3-day | -682(-2390,1026) | -1010(-2975,955) | 246(-2956,3448) | 0.502 |
|  | 5-day | -902(-3279,1475) | -1388(-4169,1394) | 275(-3985,4535) | 0.511 |
| Elemental Carbon | 1-day | -1661(-3460,138) | -3213(-5285,-1141) | 1951(-1085,4986) | 0.004 |
|  | 2-day | -2053(-4104,-3) | -2749(-5021,-478) | 621(-3637,4878) | 0.161 |
|  | 3-day | -1225(-3099,649) | -1673(-3766,419) | 412(-3365,4189) | 0.33 |
|  | 5-day | -1633(-4009,743) | -2262(-4900,376) | 836(-4227,5900) | 0.279 |
| Organic Carbon | 1-day | -1261(-5205,2684) | -2451(-7221,2319) | 1187(-5760,8134) | 0.393 |
|  | 2-day | -805(-5541,3930) | -835(-6492,4822) | -856(-9312,7600) | 0.997 |
|  | 3-day | 434(-4229,5097) | 458(-5174,6090) | 307(-7879,8494) | 0.976 |
|  | 5-day | 2256(-2457,6970) | 2112(-3667,7890) | 2480(-5707,10667) | 0.942 |
| NO_X_ | 1-day | -316(-1904,1272) | -1409(-3286,468) | 1980(-642,4601) | 0.032 |
|  | 2-day | -955(-2585,674) | -1768(-3682,145) | 955(-1892,3802) | 0.11 |
|  | 3-day | -942(-2768,884) | -1693(-3814,429) | 1040(-2307,4388) | 0.168 |
|  | 5-day | -1425(-3977,1126) | -2591(-5539,358) | 1833(-3010,6675) | 0.122 |
| Carbon Monoxide | 1-day | -1274(-3208,660) | -2190(-4350,-30) | 1641(-2020,5303) | 0.067 |
|  | 2-day | -1804(-3844,235) | -2504(-4818,-189) | 296(-3572,4164) | 0.21 |
|  | 3-day | -1195(-3365,974) | -1757(-4219,705) | 567(-3688,4822) | 0.344 |
|  | 5-day | 249(-1994,2493) | -130(-2765,2506) | 1178(-2885,5241) | 0.589 |
| O_3_ | 1-day | -1399(-3950,1152) | -848(-3776,2080) | -3094(-8258,2070) | 0.456 |
|  | 2-day | -711(-3855,2433) | -66(-3714,3582) | -2582(-8831,3668) | 0.495 |
|  | 3-day | -438(-3469,2594) | 322(-3207,3850) | -2545(-8407,3318) | 0.409 |
|  | 5-day | 905(-2241,4052) | 2358(-1320,6036) | -2676(-8372,3019) | 0.138 |
| Particle Mass |  |  |  |  |  |
| PM_0.25_ | 1-day | -1572(-3737,592) | -2200(-4886,485) | -498(-3954,2958) | 0.434 |
|  | 2-day | -1405(-3639,830) | -1721(-4564,1121) | -935(-4501,2630) | 0.733 |
|  | 3-day | -1514(-4379,1350) | -1884(-5406,1639) | -873(-5662,3917) | 0.735 |
|  | 5-day | -1468(-4754,1819) | -1670(-5451,2111) | -988(-6965,4988) | 0.845 |
| PM_0.25-2.5_ | 1-day | -260(-1223,703) | -209(-1225,807) | -734(-3792,2325) | 0.749 |
|  | 2-day | -193(-1666,1279) | 95(-1503,1692) | -1821(-5619,1977) | 0.361 |
|  | 3-day | -32(-2081,2017) | 381(-1892,2654) | -1814(-6538,2909) | 0.41 |
|  | 5-day | -363(-2116,1390) | -9(-2073,2055) | -1292(-4631,2047) | 0.521 |
| PM_2.5-10_ | 1-day | -45(-1426,1335) | -423(-2037,1191) | 863(-1555,3282) | 0.369 |
|  | 2-day | -612(-2469,1245) | -926(-3140,1287) | 82(-3131,3294) | 0.603 |
|  | 3-day | -370(-2700,1960) | -736(-3522,2050) | 401(-3587,4389) | 0.637 |
|  | 5-day | -703(-2317,912) | -946(-2917,1024) | -213(-3012,2586) | 0.672 |
| 5-day composite components | |  |  |  |  |
| PAH |  | -297(-2043,1448) | -608(-2668,1452) | 524(-2750,3799) | 0.564 |
| Organic Acids |  | -17(-1417,1382) | 130(-1482,1742) | -479(-3322,2364) | 0.714 |
| WSOC |  | 174(-1632,1980) | 382(-1708,2473) | -370(-3639,2900) | 0.695 |
| Macrophage ROS |  |  |  |  |  |
| PM_0.25-2.5_ |  | -1314(-3744,1115) | -861(-3825,2103) | -2215(-6367,1937) | 0.599 |
| PM_0.25_ |  | -69(-1408,1269) | -135(-1827,1558) | 46(-2110,2203) | 0.896 |

**Table S3 (cont.)** **Associations of CRP, IL-6sR and sTNFRII with Outdoor Air Pollutants**

|  |  | **sTNFRII** (95% CI)^a^ | | | **Interaction p-value**^b^ |
| --- | --- | --- | --- | --- | --- |
| **Air Pollutant Exposures** | | **All Subjects** | **Haplogroup H** | **Haplogroup U** |  |
| Black Carbon | 1-day | 23(-51,97) | 56(-32,144) | -42(-161,77) | 0.169 |
|  | 2-day | 25(-52,102) | 60(-29,150) | -61(-197,75) | 0.133 |
|  | 3-day | 43(-49,135) | 74(-31,180) | -43(-213,128) | 0.24 |
|  | 5-day | 23(-103,149) | 66(-82,214) | -80(-305,145) | 0.278 |
| Elemental Carbon | 1-day | -12(-103,79) | 1(-105,107) | -42(-197,113) | 0.636 |
|  | 2-day | 0(-101,102) | 26(-85,138) | -106(-321,110) | 0.272 |
|  | 3-day | 63(-35,160) | 79(-30,188) | 5(-194,203) | 0.509 |
|  | 5-day | 69(-56,194) | 80(-59,219) | 29(-241,298) | 0.735 |
| Organic Carbon | 1-day | 24(-203,252) | 25(-249,299) | 27(-366,420) | 0.993 |
|  | 2-day | 34(-237,305) | 59(-264,381) | -15(-508,477) | 0.804 |
|  | 3-day | 143(-139,425) | 178(-159,515) | 68(-439,575) | 0.721 |
|  | 5-day | 141(-149,431) | 143(-208,494) | 138(-364,640) | 0.987 |
| NO_X_ | 1-day | 53(-35,142) | 89(-16,195) | -15(-157,127) | 0.228 |
|  | 2-day | 31(-58,121) | 64(-43,170) | -36(-188,116) | 0.278 |
|  | 3-day | 59(-44,161) | 74(-45,193) | 25(-158,208) | 0.651 |
|  | 5-day | 51(-94,197) | 68(-102,237) | 15(-253,284) | 0.742 |
| Carbon Monoxide | 1-day | 72(-39,182) | 103(-23,228) | -17(-218,184) | 0.3 |
|  | 2-day | 50(-66,166) | 86(-47,219) | -45(-254,164) | 0.283 |
|  | 3-day | 77(-43,197) | 92(-45,229) | 34(-196,265) | 0.667 |
|  | 5-day | 107(-47,261) | 138(-44,319) | 34(-241,309) | 0.529 |
| O_3_ | 1-day | 14(-133,160) | 22(-146,191) | -13(-309,283) | 0.839 |
|  | 2-day | 6(-176,188) | -23(-235,190) | 90(-268,449) | 0.593 |
|  | 3-day | 6(-169,180) | -25(-229,179) | 90(-245,425) | 0.564 |
|  | 5-day | 15(-164,194) | -24(-236,187) | 103(-213,418) | 0.503 |
| Particle Mass |  |  |  |  |  |
| PM_0.25_ | 1-day | 28(-78,133) | 30(-101,161) | 25(-142,193) | 0.964 |
|  | 2-day | 6(-102,113) | 9(-128,146) | 1(-169,171) | 0.94 |
|  | 3-day | 33(-105,171) | 32(-138,203) | 34(-191,259) | 0.992 |
|  | 5-day | 42(-143,228) | 49(-165,262) | 27(-303,357) | 0.909 |
| PM_0.25-2.5_ | 1-day | -7(-59,45) | 2(-53,56) | -89(-255,76) | 0.304 |
|  | 2-day | 1(-78,81) | 20(-66,106) | -108(-315,100) | 0.264 |
|  | 3-day | -21(-131,89) | 8(-114,130) | -149(-405,107) | 0.275 |
|  | 5-day | -28(-124,68) | -13(-125,100) | -67(-252,117) | 0.618 |
| PM_2.5-10_ | 1-day | -20(-92,51) | -20(-104,64) | -20(-147,106) | 0.997 |
|  | 2-day | -38(-130,54) | -30(-140,79) | -53(-213,107) | 0.814 |
|  | 3-day | 0(-115,116) | 0(-138,137) | 1(-197,199) | 0.99 |
|  | 5-day | -40(-129,48) | -33(-141,74) | -56(-210,98) | 0.815 |
| 5-day composite components | |  |  |  |  |
| PAH |  | 66(-32,163) | 63(-53,178) | 78(-104,260) | 0.888 |
| Organic Acids |  | -96(-190,-2) | -78(-186,30) | -148(-339,42) | 0.528 |
| WSOC |  | 64(-35,163) | 43(-72,158) | 117(-60,294) | 0.477 |
| Macrophage ROS |  |  |  |  |  |
| PM_0.25-2.5_ |  | -35(-167,98) | -34(-196,127) | -37(-263,190) | 0.985 |
| PM_0.25_ |  | 80(6,154) | 50(-43,144) | 129(11,246) | 0.302 |

**^a^** Regression coefficients and 95% confidence intervals are for the expected change in the blood plasma biomarker associated with an interquartile range change in the air pollutant (see Table 2). All models are adjusted for temperature of the same averaging time, and exposures are mean centered by study phase and group.

**^b^** Stratified results for mitochondrial haplogroup are derived from product term (interaction) models of the pollutant by haplogroup H/U.
