# Supplementary material for: Mitochondrial Genetic Background Modifies the Relationship between Traffic-Related Air Pollution Exposure and Systemic Biomarkers of Inflammation
Source: PLoS One. 2013 May 23;8(5):e64444. doi: 10.1371/journal.pone.0064444 (PMC3662686; doi:10.1371/journal.pone.0064444)
Supplement: Figure S1 — Associations of biomarkers with traffic-related air pollutants for haplogroups H, U and Other (DOCX) [file pone.0064444.s005.docx]

**Figure S1.** Associations of biomarkers with traffic-related air pollutants: effect modification by mitochondrial haplogroups H, U and Other. Expected change in IL-6 (A) and TNF-α (B) (coefficient and 95% CI) corresponds to an IQR increase in air pollutant exposure (Table 2, main text).

p-value for interaction between haplogroups U and Other: ^*^p≤0.1. ^**^p≤0.05. ^#^p≤0.01.
